# Motivators and Demotivators for COVID-19 Vaccination Based on Co-Occurrence Networks of Verbal Reasons for Vaccination Acceptance and Resistance: Repetitive Cross-Sectional Surveys and Network Analysis

**DOI:** 10.2196/50958

**Published:** 2024-04-22

**Authors:** Qiuyan Liao, Jiehu Yuan, Irene Oi Ling Wong, Michael Yuxuan Ni, Benjamin John Cowling, Wendy Wing Tak Lam

**Affiliations:** 1 School of Public Health Li Ka Shing Faculty of Medicine The University of Hong Kong Hong Kong China (Hong Kong); 2 World Health Organization Collaborating Center for Infectious Disease Epidemiology and Control School of Public Health Li Ka Shing Faculty of Medicine, The University of Hong Kong Hong Kong China (Hong Kong); 3 State Key Laboratory of Brain and Cognitive Sciences University of Hong Kong Hong Kong China (Hong Kong); 4 Urban Systems Institute University of Hong Kong Hong Kong China (Hong Kong); 5 Laboratory of Data Discovery for Health Hong Kong Science and Technology Park Hong Kong China (Hong Kong); 6 Jockey Club Institute of Cancer Care Li Ka Shing Faculty of Medicine, University of Hong Kong Hong Kong China (Hong Kong)

**Keywords:** COVID-19, vaccination acceptance, vaccine hesitancy, motivators, co-occurrence network analysis

## Abstract

**Background:**

Vaccine hesitancy is complex and multifaced. People may accept or reject a vaccine due to multiple and interconnected reasons, with some reasons being more salient in influencing vaccine acceptance or resistance and hence the most important intervention targets for addressing vaccine hesitancy.

**Objective:**

This study was aimed at assessing the connections and relative importance of motivators and demotivators for COVID-19 vaccination in Hong Kong based on co-occurrence networks of verbal reasons for vaccination acceptance and resistance from repetitive cross-sectional surveys.

**Methods:**

We conducted a series of random digit dialing telephone surveys to examine COVID-19 vaccine hesitancy among general Hong Kong adults between March 2021 and July 2022. A total of 5559 and 982 participants provided verbal reasons for accepting and resisting (rejecting or hesitating) a COVID-19 vaccine, respectively. The verbal reasons were initially coded to generate categories of motivators and demotivators for COVID-19 vaccination using a bottom-up approach. Then, all the generated codes were mapped onto the 5C model of vaccine hesitancy. On the basis of the identified reasons, we conducted a co-occurrence network analysis to understand how motivating or demotivating reasons were comentioned to shape people’s vaccination decisions. Each reason’s eigenvector centrality was calculated to quantify their relative importance in the network. Analyses were also stratified by age group.

**Results:**

The co-occurrence network analysis found that the perception of personal risk to the disease (egicentrality=0.80) and the social responsibility to protect others (egicentrality=0.58) were the most important comentioned reasons that motivate COVID-19 vaccination, while lack of vaccine confidence (egicentrality=0.89) and complacency (perceived low disease risk and low importance of vaccination; egicentrality=0.45) were the most important comentioned reasons that demotivate COVID-19 vaccination. For older people aged ≥65 years, protecting others was a more important motivator (egicentrality=0.57), while the concern about poor health status was a more important demotivator (egicentrality=0.42); for young people aged 18 to 24 years, recovering life normalcy (egicentrality=0.20) and vaccine mandates (egicentrality=0.26) were the more important motivators, while complacency (egicentrality=0.77) was a more important demotivator for COVID-19 vaccination uptake.

**Conclusions:**

When disease risk is perceived to be high, promoting social responsibility to protect others is more important for boosting vaccination acceptance. However, when disease risk is perceived to be low and complacency exists, fostering confidence in vaccines to address vaccine hesitancy becomes more important. Interventions for promoting vaccination acceptance and reducing vaccine hesitancy should be tailored by age.

## Introduction

### Background

Despite strong evidence on the effectiveness of COVID-19 vaccines for preventing severe illness, hospitalization, and deaths associated with COVID-19 [1-3], vaccine hesitancy remains widespread and an important barrier for achieving high population uptake of the vaccines [4]. Hong Kong has learned its painful lesson of low vaccination uptake from the explosive epidemic caused by the Omicron BA.2 variant in February to April 2022, which claimed >10,000 lives of its 7 million people within 3 months, the highest per capita COVID-19 mortality worldwide in 2022 [5]. The devasting impact of this outbreak was mainly attributed to the low uptake of the COVID-19 vaccination among people aged ≥60 years before the pandemic. Administration of the mRNA vaccine BNT162b2 (BioNTech, Fosun Pharma, or Pfizer) and the inactivated vaccine CoronaVac (Sinovac) began in February 2021. To boost vaccine uptake, from May to June 2021, the Hong Kong government invited the business sector to offer various vaccination incentives such as lottery prizes (“lucky draw”) and vaccination leaves [6]. The Hong Kong government also introduced the concept of “vaccine bubble” in April 2021 [7], which was regarded as a preparatory stage for the implementation of the “vaccine pass.” Under the “vaccine bubble” strategy, citizens and staff of premises were encouraged to take the COVID-19 vaccination to enjoy some relaxation of social distancing measures [7], for instance, by allowing more customers per table in the restaurants if all staff and customers were vaccinated. Despite various efforts, by December 23, 2021, shortly before Omicron was introduced into the community, only 49% of persons aged ≥60 years had received at least 2 doses of COVID-19 vaccines in Hong Kong, and only 7% had received a third dose [8], an important reason for a much higher mortality rate, particularly among older people during the Omicron outbreak. In February 2022, the “vaccine pass” was implemented, which required all eligible persons to receive at least 1 dose of the COVID-19 vaccine to gain access to specified premises [9].

The reasons why people accepted or refused the COVID-19 vaccines are multifaceted, including cultural value, social norms, perceived disease risk, confidence in vaccines, convenience, and trust in government and the health system [10,11]. In view of the complexity of vaccine hesitancy and its determinants, the Strategic Advisory Group of Experts Working Group on Vaccine Hesitancy developed the 3C model, which proposes that vaccine hesitancy is shaped by 3 categories of determinants: *complacency*, *convenience*, and *confidence* [12]. The 3C model was later extended to include 2 more Cs: *risk calculation* and *collective responsibility* [13]. This model provides a good summary of the main categories of determinants of people’s vaccination acceptance and has been widely used to understand people’s vaccination acceptance or hesitancy [14]. On the basis of the original 3C and the extended 5C models, the determinants of different categories are interconnected to shape vaccine hesitancy [12,13]. However, existing studies mainly focused on the association of vaccination acceptance with each determinant of the 5C categories [15,16]. How these determinants are interconnected in shaping the vaccination outcome (acceptance or resistance) remains underexplored. Moreover, most studies that used the 3C or 5C model measured determinants of COVID-19 vaccine hesitancy using questionnaire surveys [17-19]; this may fail to capture the diverse and evolving context-specific factors during the COVID-19 vaccination program such as vaccination incentives [20,21] and vaccine mandates [22,23]. In summary, the multiple facets of vaccine hesitancy mean that people may accept or refuse the vaccine due to multiple psychosocial reasons and the emerging vaccination policies, which has been confirmed in qualitative research [24]. However, most current research that investigated the reasons for vaccination acceptance or hesitancy mainly described the frequency of each reason [25,26] and focused on only 1 vaccination outcome (either acceptance or hesitancy) [24,27]. There is little quantitative evidence on the co-occurrence of reasons for shaping different vaccination outcomes.

Network analysis is a promising statistical technique for depicting the multiple and complex interconnections between variables within a system and has been widely applied for characterizing the networks of psychological symptoms and attitudes [28-31]. A network, which is characterized by its nodes (eg, variables) and edges (the associations between variables), offers powerful visualization for the interactions among multiple factors in shaping a phenomenon. Network analysis is more exploratory and flexible than multivariate regression models. It adopts a complex system perspective and hence assumes that any variables (eg, reasons for vaccination acceptance or resistance) within the system can potentially interconnect with each other. Network analysis for the co-occurrence of reasons for adopting a behavior can quantify the relative importance of reasons based on not only how frequently a reason works solely but also how frequently it is comentioned with other reasons for motivating a behavior [32]. This will help to identify the more important reasons and the most effective combination of motivating reasons or demotivating concerns for intervention development and risk communication design. Therefore, the network approach is promising for studying the multifaceted reasons for vaccine hesitancy and their complex interconnections to guide intervention development.

The application of network analysis in vaccine-related attitudes has recently been expanded [33-38]. However, existing studies mainly included factors that were of specific research interests from the researchers’ perspective for network analysis [33-38]. This may miss some specific concerns from the vaccine receivers’ perspective and cause misalignment between expert risk communication and lay people’s preferences or concerns, potentially rendering the intervention ineffective. In addition, existing studies mainly focused on the networks of factors that influenced willingness to accept COVID-19 vaccines [33-38]. However, determinants and their interactions that shape vaccination acceptance and resistance can be different [39-41]. For instance, while protecting oneself and others was a more salient reason for vaccination acceptance, lacking confidence in the vaccine was a more important reason for vaccination rejection [41]. Such differences can be further complicated by the variability of motivators and demotivators for vaccination acceptance across age groups. For example, younger adults were found to refuse a COVID-19 vaccine mainly due to conspiracy beliefs and lacking trust in the government [42], while the top reasons for older adults refusing a COVID-19 vaccine were their chronic condition and old age [27,40]. However, such investigations overlooked the interconnections between motivators and demotivators. Distinguishing the networks of reasons for accepting and refusing a vaccine in different age groups is important for informing the development of vaccine risk communication that is tailored to the audience’s underlying vaccine-related attitudes. Furthermore, these studies, which used the network analysis, neither investigated the reasons relating to the 5C model and the co-occurrence of reasons nor quantified their relative importance for influencing vaccine hesitancy.

### Objective

In this study, we collected participants’ diverse verbal reasons for accepting or refusing a COVID-19 vaccine using open-ended questions through population-based telephone surveys conducted in Hong Kong during the COVID-19 pandemic and categorized the reasons based on the 5C model of vaccine hesitancy for network analysis. We aimed to provide quantitative evidence on the co-occurrence of reasons and their relative importance for both vaccine acceptance and resistance, as well as how the relative importance of motivators and demotivators for COVID-19 vaccination could differ by age.

## Methods

### Participants

Repeated cross-sectional surveys were conducted on a weekly or monthly basis to monitor acceptance of the COVID-19 vaccine among general adults after the COVID-19 vaccination campaign was launched in early 2021 in Hong Kong [43-45]. In each round, we recruited Hong Kong adults aged ≥18 years using random-digital-dialed telephone interviews with a ratio of 1:1 for landlines and mobile phones. The surveys were implemented by a local survey company that had been demonstrated to have the capacity to provide high-quality population-based surveys using random digital calls. All the calls were made during working hours and nonworking hours to avoid oversampling of nonworking people. Each sampled telephone number was called up to 5 times at different times and on different days before being replaced with a new one. For each landline-based call, if there were >1 eligible member in the household, the “next birthday rule” (the person whose next birthday is closest to the survey date) was adopted to determine the person to be interviewed. Individuals with linguistic and cognitive difficulties in completing a telephone interview were excluded. The target sample size was an alternative of 500 or 1000 for each round. In each round, core study measures such as the uptake of COVID-19 vaccines and the intention to receive a COVID-19 vaccine were retained throughout, while additional study measures were rotated to maintain a feasible length of the questionnaire for a telephone interview. Data from a total of 19 survey rounds that collected reasons for accepting a COVID-19 vaccine and 9 survey rounds that collected reasons for being hesitant or resistant about taking a COVID-19 vaccine were used for this analysis. Participants’ demographic distributions were compared between the accepting group and the resistant group using the Pearson chi-square test. All the surveys included were conducted between March 2021 and July 2022. The details of each survey round can be found in Multimedia Appendix 1.

### Ethical Considerations

The study was conducted in accordance with the Declaration of Helsinki and Good Clinical Practices. As our surveys were conducted over the telephone, written informed consent was not feasible, but verbal informed consent was obtained from all participants before the interview started. The data sets did not contain any personally identifiable information. This study received ethics approval from the institutional review board (reference UW 20-095).

### Study Instruments

To explore the reasons for accepting, hesitating, or refusing a COVID-19 vaccine, in 16 survey rounds conducted between March 1, 2021, and August 20, 2021, participants who had completed at least 1 dose of the COVID-19 vaccine were asked about the major reasons that motivated them to receive the vaccine. The Hong Kong government started recommending a vaccine booster (the third vaccine dose) for general adults who had received 2 doses of COVID-19 vaccines for at least 6 months on November 23, 2021 [46]. Therefore, in 3 additional survey rounds that were conducted after this date (between December 6, 2021, and January 13, 2022), participants who had received a vaccine booster were asked about the reasons that motivated them to receive a vaccine booster. The open-ended responses from these 19 survey rounds, in which participants were asked about their reasons for taking a COVID-19 vaccine or a vaccine booster, were coded as reasons for vaccination acceptance. In 9 survey rounds conducted between December 6, 2021, and July 14, 2022, we identified resistant participants based on their vaccination status and intention. First, participants were asked about the number of doses of COVID-19 vaccines they had received. Those who had received none, 1 dose, or 2 doses were then asked about how likely they would receive the first, second, or third vaccine dose, respectively, in the next 3 months (rated on a scale from “definitely not” to “very unlikely,” “unlikely,” “unsure,” “likely,” “very likely,” and “definitely yes”). Those who answered “definitely not,” “very unlikely,” “unlikely,” and “unsure” to the vaccination intention question were defined as the resistant group. These participants were asked about the major reasons for being hesitant about or rejecting COVID-19 vaccination. The verbal responses from these 9 survey rounds in which participants were asked about the reasons for refusing the 2-dose primary COVID-19 vaccination series or a vaccine booster were coded as reasons for vaccination resistance. The vaccination resistance reasons were collected at a later phase of the vaccination program and during the Omicron wave when COVID-19–confirmed cases soared rapidly (Multimedia Appendix 2). Therefore, our data capture reasons from the most resistant group, given that the high vaccination uptake rate had been achieved in the population and the disease risk was relatively high, but participants still reported being hesitant or resistant regarding vaccination. The data collection period and number of daily COVID-19 cases are presented in Multimedia Appendix 2. The participants were asked to provide reasons that first came to mind, and then the interviewer jotted down notes of participants’ statements and asked follow-up questions of “any other reasons” to encourage participants to give >1 reason for their vaccination decision. Across all survey rounds, participants were also asked about their sociodemographics including age, sex, educational attainment, employment status, and history of chronic conditions.

### Coded Reasons for Accepting or Resisting a COVID-19 Vaccine

The participants’ verbal responses to the open-ended questions regarding reasons for vaccination acceptance and resistance were extracted and independently coded by 2 researchers (QL and JY). We combined the bottom-up and top-down approaches to generate the main categories of these qualitative verbal responses. First, basic categories were independently generated inductively by the 2 researchers by making sense of the meanings of the participants’ verbal responses (bottom-up approach). Following the initial coding, the 5C categories (complacency, confidence, convenience, calculation, and collective responsibility) of vaccine hesitancy [13] were used to guide the clustering of the initial codes to generate the main categories (top-down approach). Although the 5C model was used as a guide for generating categories of motivators and demotivators, the 2 researchers remained open to allow new categories that were not covered by the 5C model to emerge from the data. Following completion of the initial coding, each coder manually checked 10% of the codes generated by another to ensure consistency and consensus on all codes. Any inconsistencies in coding were solved by reviewing the raw data and having joint discussions between the 2 coders (QL and JY). A total of 52 responses that were coded as the reasons for vaccination acceptance and 23 responses that were coded as the reasons for vaccination resistance were excluded from the final analyses because their meanings were judged ambiguous by both coders. To facilitate interpretation, we finally mapped the coded reasons onto the 5C categories but allowed new categories to emerge. This was aimed at facilitating data interpretation and minimizing conceptual or meaning overlap between the coded reasons. For the mapping, each of the 2 coders (QL and JY) first reached a consensus on the definition of each of the 5C categories and then mapped each reason to 1 C. Any inconsistencies in mapping were solved by joint discussions and reviewing the original definitions and relevant literature. Finally, for reasons that cannot be mapped to any of the existing 5C categories, extended categories of the 5C categories would be proposed.

### Network Analysis

Network analysis was conducted separately for vaccination acceptance and vaccination resistance in Python using the Networkx Package (Python Software Foundation) [47]. We first determined the nodes included for network estimation and visualization. For either the acceptance or the resistance network, each reason category of vaccination acceptance or resistance, respectively, represents 1 node in the respective network. An edge between 2 nodes indicates that the 2 reasons were comentioned by participants. However, instead of directly modeling the frequency of comentioning, the edges were normalized using a weighted method following previous work [32]. Specifically, if participants mention only 1 reason for their vaccination acceptance or resistance, a standard weight of “1” will be assigned to that reason. If participants mention 2 reasons, each of the 2 reasons will get a standard weight of “1” because there is only 1 unique pair of reasons. If participants mention ≥3 reasons, the weight between each pair of reasons will be “1” divided by the number of pair combinations among the reasons. For instance, if 3 reasons (k_1_, k_2_, and k_3_) are comentioned by participants, the edge value for each pair of the 3 reasons (k_1_ – k_2_, k_1_ – k_3,_ or k_2_ – k_3_) would be one-third so that their total weight will still be equal to “1.” This method ensured that each participant contributed equally to the whole network, regardless of how many reasons they had mentioned for vaccination acceptance or resistance. Following this method, a symmetric adjacency matrix was constructed for both reasons of vaccination acceptance and resistance for network analysis. Therefore, if we have k reasons, then an edge can be expressed as E = {E_ij_|I,j = 0,1,...,k}, which is the *ith* row and *jth* column of the matrix. The weight of the edge will depend on the number of participants accepting or refusing a COVID-19 vaccine due to reasons *i* and *j.* The more the number of participants that comention the 2 reasons, the greater the weight of their edge will be. The adjacency matrix was then used to calculate each node’s eigenvector centrality—an index used for quantifying the relative importance of the node based on nodes’ co-occurrence patterns in the network [48]. Specifically, a higher normalized eigencentrality value indicates a relatively greater importance of the reason, which accounts for not only how frequently the node occurred (ie, reason was mentioned by participants) but also how frequently the node was linked to other influential nodes (ie, reason was comentioned with other frequently mentioned reasons). Mathematically, the eigenvector centrality can be obtained from the following equation: *AX = λX*

In this equation, *A* represents the adjacency matrix. The eigenvector *X* was calculated using 500 power iteration process, given the constant eigenvalue λ.

Following the same methodological procedure, we constructed the co-occurrence networks stratified by age subgroups and obtained eigencentrality values for each reason across age groups. A normalized eigencentrality measure can be used for direct comparisons of the relative importance of the same reasons for vaccination acceptance and resistance across different age groups.

## Results

### Participants

The survey cooperation rate was around 66.7%, ranging from 51.9% to 74.4%. Survey response rate was defined as the proportions of participants who completed the interviews against those who were contacted and eligible (Multimedia Appendix 1). Overall, in 19 survey rounds, 5559 participants provided reasons for accepting a COVID-19 vaccine, and in 9 survey rounds, 982 participants provided reasons for being resistant (including hesitant) about taking a COVID-19 vaccine; these reasons were used for reason co-occurrence network analysis. Comparisons of the demographics of participants who accepted the vaccine and those who were resistant to receiving the vaccine showed that the vaccination resistance group was more likely to be younger, have higher educational attainment, be unemployed, and have at least 1 chronic condition (Table 1).

**Table 1:** Participants’ demographic characteristics in the COVID-19 vaccination accepting group and resistant group between March 1, 2021, and July 14, 2022.

Characteristics			Accepting group (n=5559)		Resistant group (n=982)		Differences^a^, *P* value	
**Sex, n (%** **)**								.87
	Female	3103 (55.8)		551 (56.1)				
	Male	2456 (44.2)		431 (43.9)				
**Age groups (years),** **n (** **%)**								<.001
	18-24	289 (5.2)		129 (13.1)				
	25-34	584 (10.5)		206 (21)				
	35-44	946 (17)		146 (14.9)				
	45-54	1079 (19.4)		118 (12.)				
	55-64	1028 (18.5)		123 (12.5)				
	≥65	1498 (26.9)		225 (22.9)				
**Educational attainment, n (%)**								<.001
	≤Primary	711 (12.8)		130 (13.2)				
	Secondary	2555 (46)		359 (36.6)				
	≥Tertiary	2207 (39.7)		474 (48.3)				
**Employment status, n (%)**								<.001
	Employed	2773 (49.9)		476 (48.5)				
	Students, home makers, or retirees	2524 (45.3)		434 (44.2)				
	Unemployed^b^	175 (3.1)		56 (5.7)				
Chronic condition (yes), n (%)			1546 (27.8)		279 (28.4)		<.001	

^a^Differences in demographic distributions between participants who provided reasons for accepting a COVID-19 vaccine and those provided reasons for being hesitant or resistant about taking a COVID-19 vaccine.

^b^Unemployed group included unemployed persons or who reported that they were seeking for jobs at the survey time.

### Coded Categories of Reasons for Vaccination Acceptance and Resistance

On the basis of participants’ verbal responses to the questions asking for reasons for vaccination acceptance and resistance, 10 main categories of reasons for vaccination acceptance and vaccination resistance, respectively, were generated (Multimedia Appendix 3). The 10 main reasons for vaccination acceptance included *disease risk* (eg, worry about the COVID-19 risk; n=3689), *protecting others* (n=2354), *back to normal life* (n=877), *confidence in vaccines* (n=703), *vaccine mandates* (eg, complying with the vaccine pass; n=548), *progovernment* (eg, to support the government; n=140), *social norms* (eg, following significant others’ opinions; n=100), *convenience* (n=83), *incentives* (eg, “lucky draw”; n=40), and *trust in experts* (n=15). The 10 main reasons for vaccination resistance included *lack of vaccine confidence* (n=501), *complacency* (eg, perceiving no need for vaccination or low risk of disease; n=376), *poor health status* (eg, “I have chronic diseases”; n=116), *vaccine mandates* (eg, opposing the vaccine pass; n=52), *distrust in government* (n=27), lack of social support (eg, “no one take me to the vaccination site”; n=18), *no incentives* (eg, no “lucky draw”; n=12), *inconvenience* (n=11), *social norms* (eg, “friends/family advised me not to take the vaccination”; n=7), and *medical preference* (eg, preferring Chinese medicine; n=6).

The coded reasons are mapped onto the 5C categories of vaccine hesitancy in Table 2. Both coders decided that 3 reasons including *incentives*, *vaccine mandates*, and *medical preference* were more specific to the contexts of COVID-19 vaccination and the underlying values, hence one more “C”—context was included to cover these reasons. Some reasons were mapped to >1 C to accommodate the multiple psychological antecedents of the reasons. For instance, *poor health status* as a reason for rejecting vaccination was mapped to both *lack of vaccine confidence* and *calculation* because it refers to not only the concern about vaccine side effects but also a trade-off between the vaccine side effects and the capability of their physical body to endure the side effects.

**Table 2:** Thematic coding and mapping the reasons for COVID-19 vaccination acceptance and resistance using an extensive 5Cs of vaccine hesitancy model.

Coded reasons		Complacency	Confidence	Convenience	Calculation	Collective responsibility	Context^a^
**Reasons for vaccination acceptance**							
	(Perceived high) disease risk	✓			✓		
	Social norms	✓					
	Back to normal life	✓			✓		
	(High) confidence in vaccines		✓				
	Progovernment		✓				
	Trust in experts		✓				
	Convenience			✓			
	Protecting others					✓	
	Vaccine mandates		✓				✓
	Incentives				✓		✓
**Reasons for vaccination resistance**							
	Complacency (low disease risk, no need, and no urgency)	✓					
	(Negative) social norms	✓					
	Lack of social support	✓		✓			
	Lack of vaccine confidence		✓		✓		
	Poor health status		✓		✓		
	Distrust in government		✓				
	Inconvenience			✓			
	Medical preference (dislike vaccination)		✓				
	(Dislike) vaccine mandates		✓				✓
	No incentives				✓		✓

^a^an addition C (*context*) was included to accommodate *vaccine mandates*, *incentives*, and *medical preference*.

### Reason Co-Occurrence Network for Vaccination Acceptance

**Figure 1:**
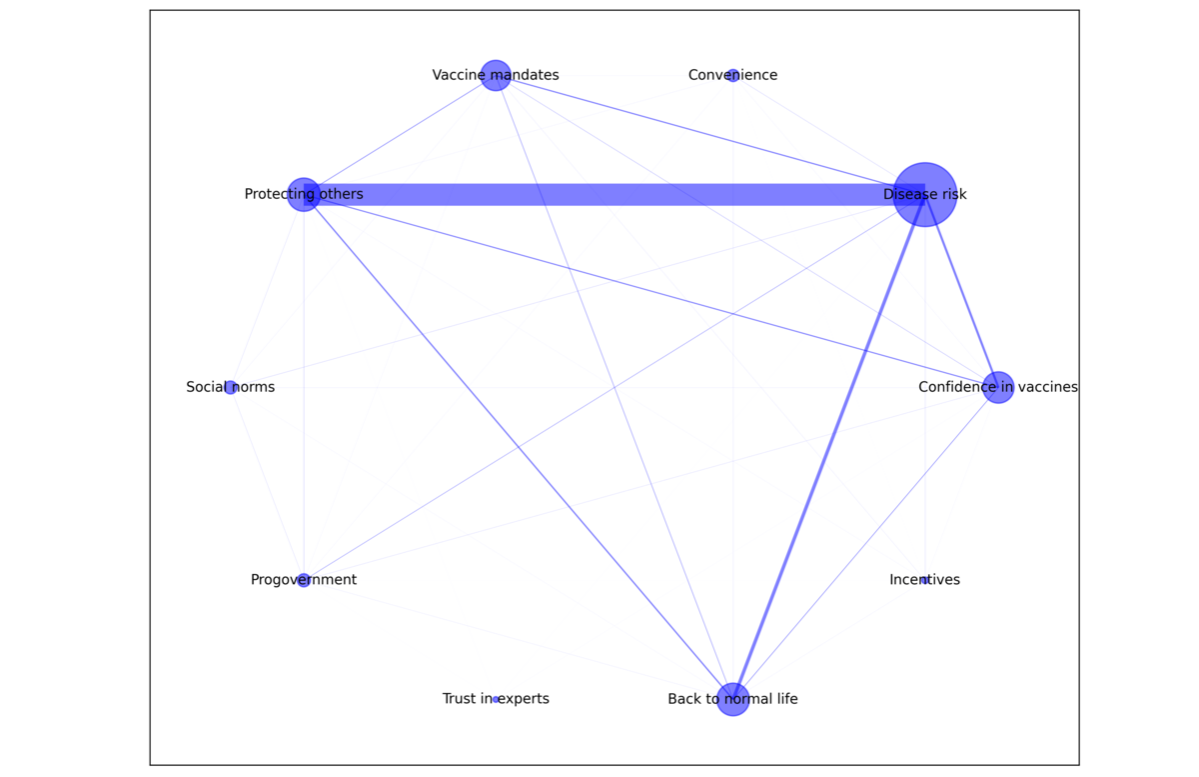
Co-occurrence network reasons for COVID-19 vaccination acceptance and their normalized eigencentrality values provided by 5559 participants between March 1, 2021, and January 13, 2022. Larger node size indicates that the reason is more frequently mentioned as a single reason for resisting the vaccine; more thickness of the edge indicates that the 2 reasons were more frequently comentioned as reasons for accepting the vaccine.

Figure 1 shows the reason co-occurrence network for vaccination acceptance. Among the 10 accepting reasons, *disease risk* followed by *protecting others* were the most important motivators with high egicentrality values (*disease risk*=0.80; *protecting others*=0.58). These 2 reasons were also the most frequently comentioned pair of reasons. The third most frequently mentioned reason by the group who accepted vaccination was *back to normal life (0.12)*. A higher co-occurrence frequency was also identified among *disease risk*, *protecting others*, *back to normal life*, *confidence in vaccines*, and *vaccine mandates*, suggesting strong interconnections among these 5 motivators in the network. *Convenience*, *incentives*, and *trust* in experts were mainly mentioned as single motivators for vaccination acceptance.

**Figure 2:**
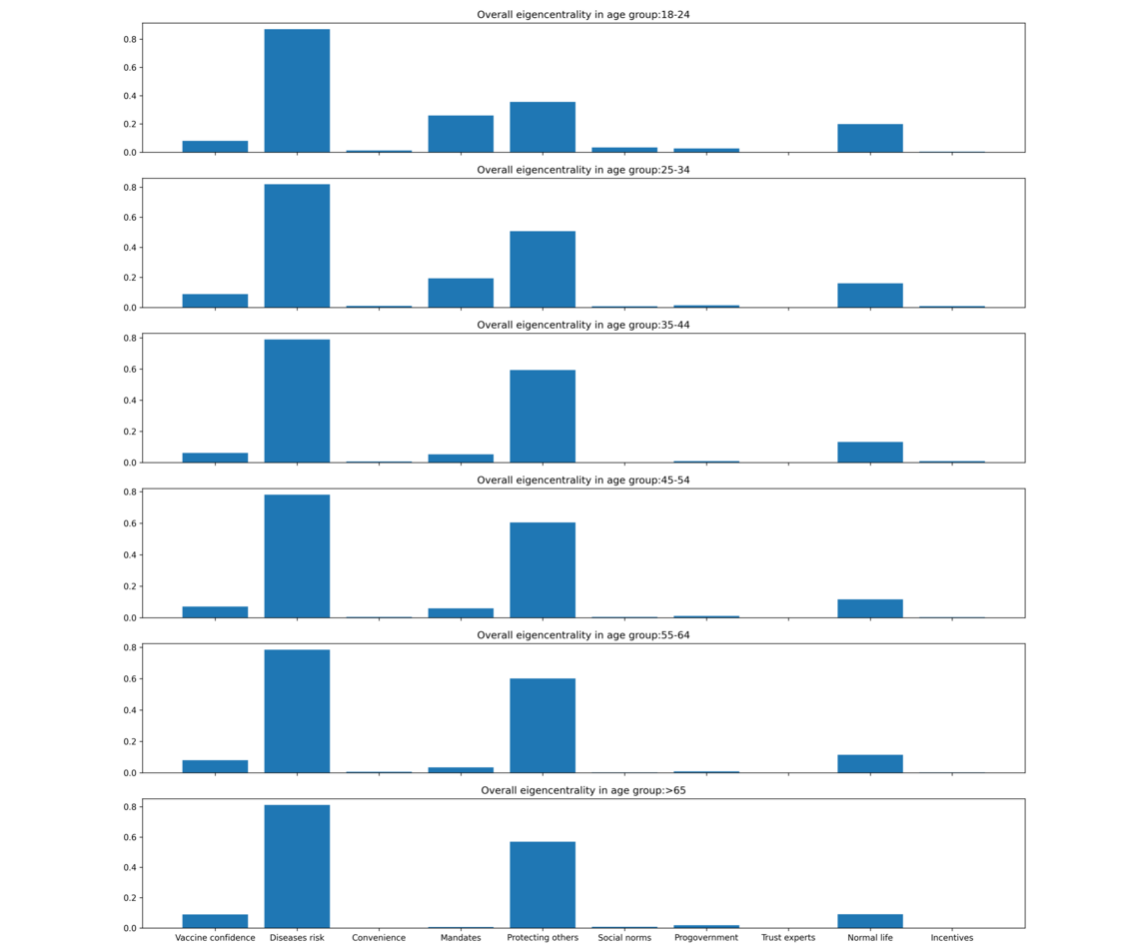
Normalized eigencentrality values of different motivators by different age groups for COVID-19 vaccination provided by 5559 participants between March 1, 2021, and January 13, 2022.

Across age subgroups, the importance of *vaccination mandates* (0.26) and *back to normal* *life* (0.20) was greater for younger people, while the importance of *protecting others* (0.57) was greater for older people for motivating vaccination acceptance (Figure 2). Mean eigencentrality value for each accepting reason across age groups are provided in a table in Multimedia Appendix 4.

### The Network for Reasons of Vaccination Resistance

**Figure 3:**
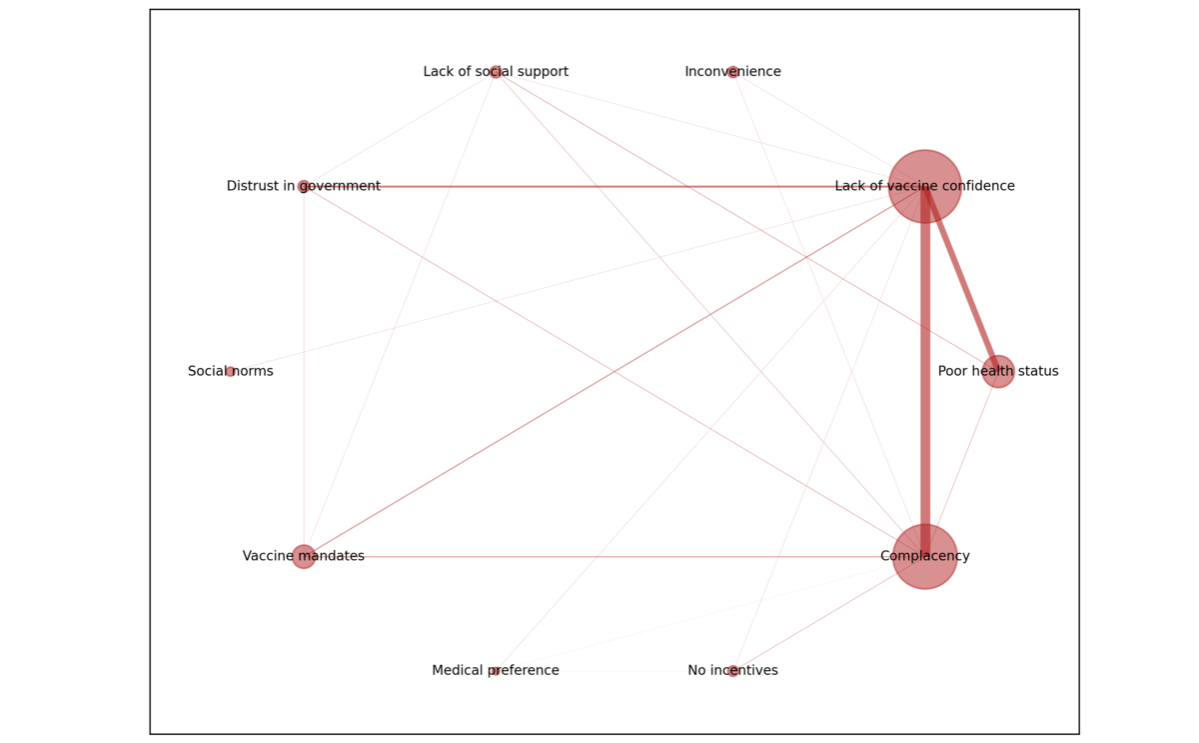
Co-occurrence network of reasons for COVID-19 vaccination resistance and their normalized eigencentrality values provided by 982 participants between December 6, 2021, and July 14, 2022. Larger node size indicates that the reason is more frequently mentioned as a single reason for resisting the vaccine; more thickness of the edge indicates that the 2 reasons were more frequently comentioned as reasons for resisting the vaccine.

Figure 3 shows the reason co-occurrence network for vaccination resistance. *Lack of vaccine confidence* (egicentrality=0.89) followed by *complacency* (egicentrality=0.45) were the most important reasons for vaccination resistance. *Poor health status* (0.09) was also a commonly cited reason among individuals with resistant attitudes toward vaccination, ranking as the third most prominent factor. Lack of vaccine confidence was most frequently comentioned with *complacency*, followed by *poor health status*, *distrust in government*, and *dislike of vaccine mandates* as demotivators of COVID-19 vaccination. These 5 demotivators formed the central part of the vaccine resistance network. *Inconvenience*, *incentives*, and *medical preference* (eg, dislike medical intervention including vaccination) were mostly given as the sole reasons for vaccination resistance by participants.

**Figure 4:**
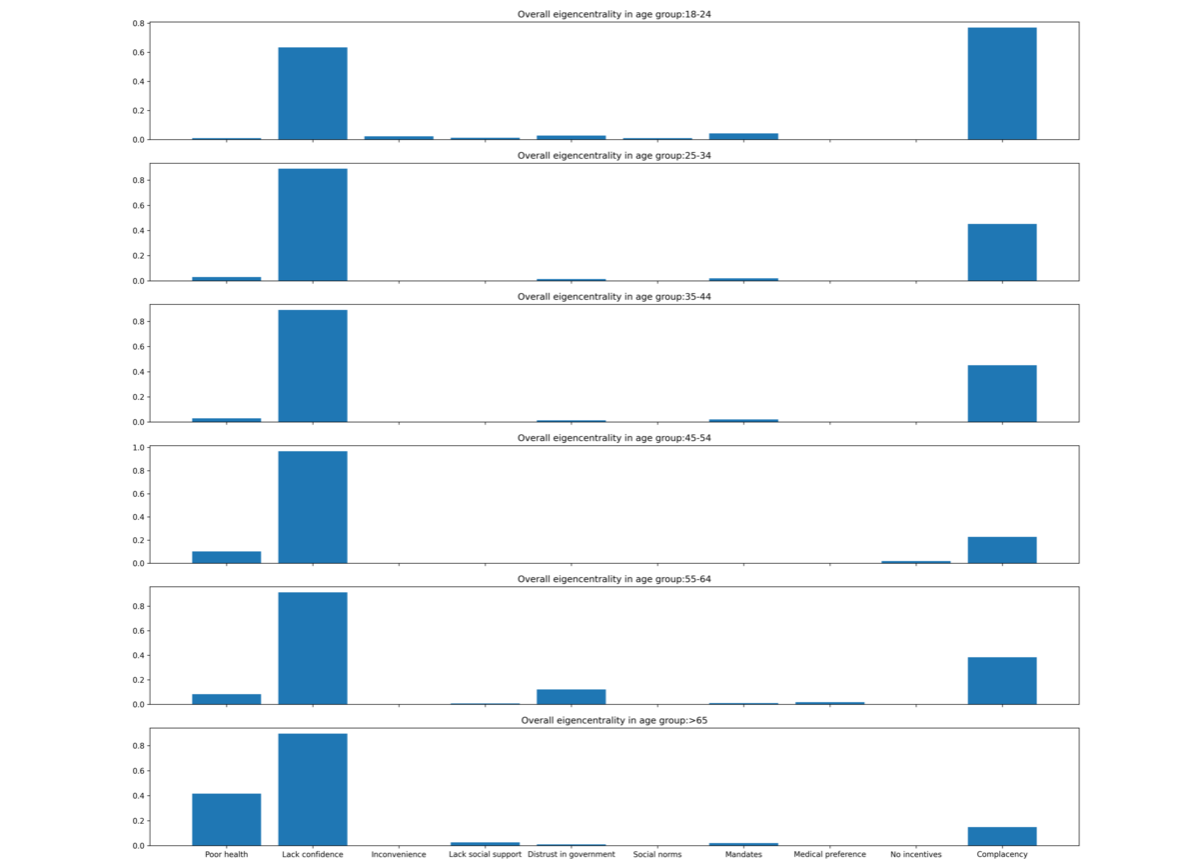
Normalized eigencentrality values of demotivators by different age groups for COVID-19 vaccination provided by 982 participants between December 6, 2021, and July 14, 2022.

Across age subgroups, *complacency* and *poor health status* were important reasons for younger and older people, respectively, for resisting vaccination (Figure 4). Mean eigencentrality value for each acceptant reason across age groups was provided in Multimedia Appendix 4.

## Discussion

### Principal Findings

We used open-ended questions to elicit more nuanced and richer responses from participants to investigate the reasons for COVID-19 vaccination acceptance and resistance. We linked their verbal reasons to the 5Cs of vaccine hesitancy to improve the theoretical relevance of the findings. Most reasons for vaccination acceptance and resistance can be mapped to the 5Cs: complacency, confidence, convenience, calculation, and collective responsibility. However, one more C, namely, *context*, was included to accommodate *vaccine mandates* and *incentives*, which represent specific contexts of COVID-19 vaccination.

The reason co-occurrence networks showed that while *disease risk* (lack of complacency) and *protecting others* (collective or social responsibility) were the most important reasons for vaccination acceptance, *lack of vaccine confidence* and *complacency* (ie, perceived low disease risk and perceived low importance of vaccination) were the most important reasons for vaccination resistance. Overall, a comparison of the networks for vaccination acceptance and resistance indicates that when *disease risk* and *protecting others* are salient for motivating vaccination uptake, *confidence* in vaccines becomes less important. This is consistent with previous findings that perceiving high personal risk of the pandemic could override people’s concern about the uncertain safety of new vaccines in vaccination decision-making [49]. However, our study indicates that when disease risk is perceived to be low and complacency exists, *lack of vaccine confidence* becomes the dominant reason for demotivating vaccination uptake. This explains why merely communicating vaccine benefits (eg, protection of oneself or others) may have a minimal effect on vaccine uptake when people perceive a low risk of the disease, particularly among those who are at low risk for severe consequences of the disease [50].

For vaccination acceptance, *protecting others* was frequently comentioned with *disease risk* as a motivator for vaccination uptake, suggesting a strong connection between the perceived risk of the disease to themselves and the social responsibility to protect others [51]. Prosocial vaccination (vaccination for protecting others) has been suggested to be an important messaging strategy for promoting COVID-19 vaccination acceptance [52,53]. Our study suggests that prosocial interventions should be combined with messages for promoting perceived personal risk of the disease to enhance its persuasive effects. The age-specific network indicated that *protecting others* was a more important motivator for older people than it was for younger people, although theoretically, younger people should be more likely to take vaccinations to protect others because the severity of COVID-19 increased with age [54]. Vaccinating the younger population against COVID-19 to block the transmission of SARS-CoV-2 in the community was a common advocacy for promoting the young population’s vaccination uptake [55]. However, our results suggest that older people value social responsibility more when making vaccination decisions, which was also identified in other contexts [56,57]. A previous study found that communicating prosocial benefits is effective in increasing multiple vaccination uptake in older adults [58]. Future interventions targeted at this age group should highlight the prosocial benefits and community responsibility to motivate older adults’ vaccination uptake.

Recovering life normalcy was the third important motivator for COVID-19 vaccination acceptance and had a high frequency of co-occurrence with *disease risk* and *protecting others*. The COVID-19 vaccination or the vaccine pass had been frequently framed as a strategy to restore “new normalcy,” that is, relaxation of social distancing measures once high vaccination uptake of the population is achieved [59]. However, our study suggests that people’s vaccination motivation of returning to life normalcy is not a salient motivator; it could be simultaneously correlated with their understanding of the disease risk to themselves and others. A recent experimental study found that messages that comention the private health benefit and economic benefit (eg, vaccines enable economic recovery) can increase vaccination intention by 9% compared with the control group [60]. Future vaccination communication can highlight the cobenefits of returning life to normalcy and protecting oneself and others to achieve optimal effect.

Vaccine mandates is another factor in the context domain; it can be either a motivator or a demotivator of vaccination uptake. Although the “vaccine bubble” was framed as a benefit of vaccination for granting people greater life normalcy [7], it was also perceived to be a constraint on people’s freedom in vaccination choice, which impaired public trust in authorities [61,62]. In our coding, we combined “vaccine bubble” and “vaccine pass” as 1 code, namely, “vaccine mandates.” Our study showed that *vaccine mandates* as a vaccination motivator was connected to *disease risk* and *protecting others*, but as a vaccination demotivator, it was connected to *lack of vaccine confidence*, *complacency*, and *distrust* in government. This indicates that whether *vaccine mandates* act as a motivator or demotivator depends on individuals’ perceived disease risk, confidence in vaccine, and trust in the government. This finding provides practical implications for how to frame vaccine mandates in a vaccination campaign; that is, vaccine mandates, when framed as a strategy for self-protection and prosocial values by highlighting disease risk and a way for themselves and others to enjoy normal social activities, could motivate vaccination uptake. However, framing vaccine mandates as a coercive measure may be regarded as freedom violation and demotivate vaccination uptake [63]. Both *life normalcy* and *vaccine mandates* were more important for younger people as motivators for vaccination acceptance than they were for older people, and hence should be the important intervention targets for promoting younger people’s vaccination uptake in the future.

For vaccination resistance, complacency was strongly connected with *lack of vaccine confidence*, suggesting that when disease risk is perceived to be low, uncertain vaccine safety can become salient in hindering vaccination uptake [49]. *Complacency* was a more important demotivator for younger people than it was for older people, which is consistent with the fact that COVID-19 is a less severe disease for younger people [54]. *Poor health status* was the third important demotivator for COVID-19 vaccination, which was strongly connected with *lack of vaccine confidence* in the network. Such a pattern was more prevalent in older people, suggesting that the poor health concern mainly comprises concern about vaccine side effects or safety in this group. One previous qualitative study suggested that older people who perceived their health status was poor due to aging or chronic diseases tended to perceive themselves to have lower capability to endure the vaccine side effects [40]. Such perception was linked to their value of aging and lack of social support [40]. This highlights the importance of addressing people’s concerns about their health status for addressing vaccine hesitancy through enhancing confidence in vaccines, reshaping the value of aging, and enhancing social support [64].

Both the networks for vaccination acceptance and resistance showed that convenience (or inconvenience) and incentives (or no incentives) mainly represented as single reasons for motivating or demotivating COVID-19 vaccination uptake. In Hong Kong, convenience in accessing the COVID-19 vaccine had been greatly increased by setting up multiple vaccination sites, extending service hours for vaccination services, and offering the vaccines free for all [46]. In addition, the government offered vaccination leaves and relaxation of social distancing measures, while a series of lucky draws were sponsored by the business sector as reward strategies to boost vaccination uptake. Although convenience and incentives (or perceiving no convenience or incentives) can serve as independent cues or heuristics to motivate or demotivate vaccination uptake [65], the low frequency of these reasons and their weak connections with other reasons indicate that they have limited and mainly transient effects on COVID-19 vaccination uptake [66].

### Limitations

Our study had several limitations. First, although participants were encouraged to give >1 reason for accepting, refusing, or being hesitant about taking COVID-19 vaccination, the reasons for COVID-19 vaccination acceptance and resistance could not be exhaustively explored through a telephone survey. We assumed that the reasons mentioned by participants were more mentally accessible for them and thereby more salient in influencing their vaccination decision. Nevertheless, there is still a possibility that some underlying reasons for vaccination acceptance or resistance were hidden by the participants, consciously or unconsciously. Second, our network analysis focused on the co-occurrence of reasons but could not determine the causal relationships between the reasons. Third, although 2 coders independently coded the reasons to ensure the reliability of coding, there remains uncertainty in making sense of participants’ verbal responses and the combination of reasons into main categories. Despite this, we strictly documented the procedure and ensured transparency of the decision-making process to improve the trustworthiness of our findings.

### Conclusions

Perception of personal risk to disease and the social responsibility to protect others were the most important comotivators, while lack of vaccine confidence and complacency were the most important co-demotivators for COVID-19 vaccination. For COVID-19 vaccination acceptance, recovering life normalcy, confidence in vaccines, and vaccine mandates were additional motivators, but these reasons were likely to work based on people’s understanding of the disease risk to themselves and others. For vaccination resistance, perception of poor health status, distrust in the government, and dislike of vaccine mandates were additional demotivators, all of which were linked to lack of vaccine confidence and complacency. Convenience and immediate incentives for vaccination were mainly mentioned as the single reasons for accepting or resisting the COVID-19 vaccination. For older people, protecting others was a more important motivator, while perception of poor health status was a more important demotivator. For younger people, recovering normal life and vaccine mandates were more important motivators, while complacency was a more important demotivator for the COVID-19 vaccination.

## Appendix

Multimedia Appendix 1 An overview of study survey rounds.

Multimedia Appendix 2 Data collection period and number of daily COVID-19 cases.

Multimedia Appendix 3 Coded categories of reasons for COVID-19 vaccination acceptance and resistance.

Multimedia Appendix 4 Mean eigencentrality values of vaccination acceptance and resistance reasons by age groups.

## Data Availability

The data set generated during and analyzed during this study is available from the corresponding author on reasonable request.
