# Supplementary material for: Motivators and Demotivators for COVID-19 Vaccination Based on Co-Occurrence Networks of Verbal Reasons for Vaccination Acceptance and Resistance: Repetitive Cross-Sectional Surveys and Network Analysis
Source: JMIR Public Health Surveill. 2024 Apr 22;10:e50958. doi: 10.2196/50958 (PMC11074890; doi:10.2196/50958)
Supplement: Multimedia Appendix 1 [file publichealth_v10i1e50958_app1.docx]

Multimedia Appendix Table 1. An overview of study survey rounds

| Timeframe | Sample size, cooperation rate ^a^ (N, %) | Measured acceptant reasons | Measured resistant reasons |
| --- | --- | --- | --- |
| 2021 | | | |
| Mar 1 - 3 | 1007 (72.0) | ✓ |  |
| Mar 22 - 25 | 502 (62.2) | ✓ |  |
| Mar 29 - 31 | 1001 (64.6) | ✓ |  |
| Apr 12 - 15 | 1015 (68.9) | ✓ |  |
| Apr 19 - 22 | 502 (64.9) | ✓ |  |
| Apr 27 -30 | 1010 (66.6) | ✓ |  |
| May 3 - 7 | 503 (71.0) | ✓ |  |
| May 10 - 14 | 1000 (67.7) | ✓ |  |
| May 17 - 21 | 500 (69.1) | ✓ |  |
| May 24 - 28 | 1003 (62.3) | ✓ |  |
| May 31 – Jun 6 | 513 (69.2) | ✓ |  |
| Jun 21 - 25 | 1004 (71.2) | ✓ |  |
| Jul 5 - 9 | 1006 (72.5) | ✓ |  |
| Jul 19 - 24 | 1005 (68.1) | ✓ |  |
| Aug 3 - 6 | 1007 (71.7) | ✓ |  |
| Aug 16 - 20 | 1006 (68.0) | ✓ |  |
| Dec 6 - 16 | 1026 (70.0) | ✓ | ✓ |
| Dec 20 - 30 | 1014 (71.0) | ✓ | ✓ |
| 2022 | | | |
| Jan 3 -13 | 1013 (74.4) | ✓ | ✓ |
| Mar 28 – 31 | 504 (68.6) |  | ✓ |
| Apr 4 – 14 | 1014 (51.9) |  | ✓ |
| Apr 19 – 28 | 1001 (58.8) |  | ✓ |
| May 3 - 12 | 1007 (62.3) |  | ✓ |
| Jun 6 -15 | 1003 (59.7) |  | ✓ |
| Jul 5 - 14 | 1019 (61.5) |  | ✓ |

^a^ Survey cooperation rate is defined as the proportions of participants who completed the interviews against subjects who were contacted and identified to be eligible.
