# Supplementary figures and images for: Motivators and Demotivators for COVID-19 Vaccination Based on Co-Occurrence Networks of Verbal Reasons for Vaccination Acceptance and Resistance: Repetitive Cross-Sectional Surveys and Network Analysis
Source: JMIR Public Health Surveill. 2024 Apr 22;10:e50958. doi: 10.2196/50958 (PMC11074890; doi:10.2196/50958)

Appendix Figure 1. Data collection period and number of daily COVID-19 cases


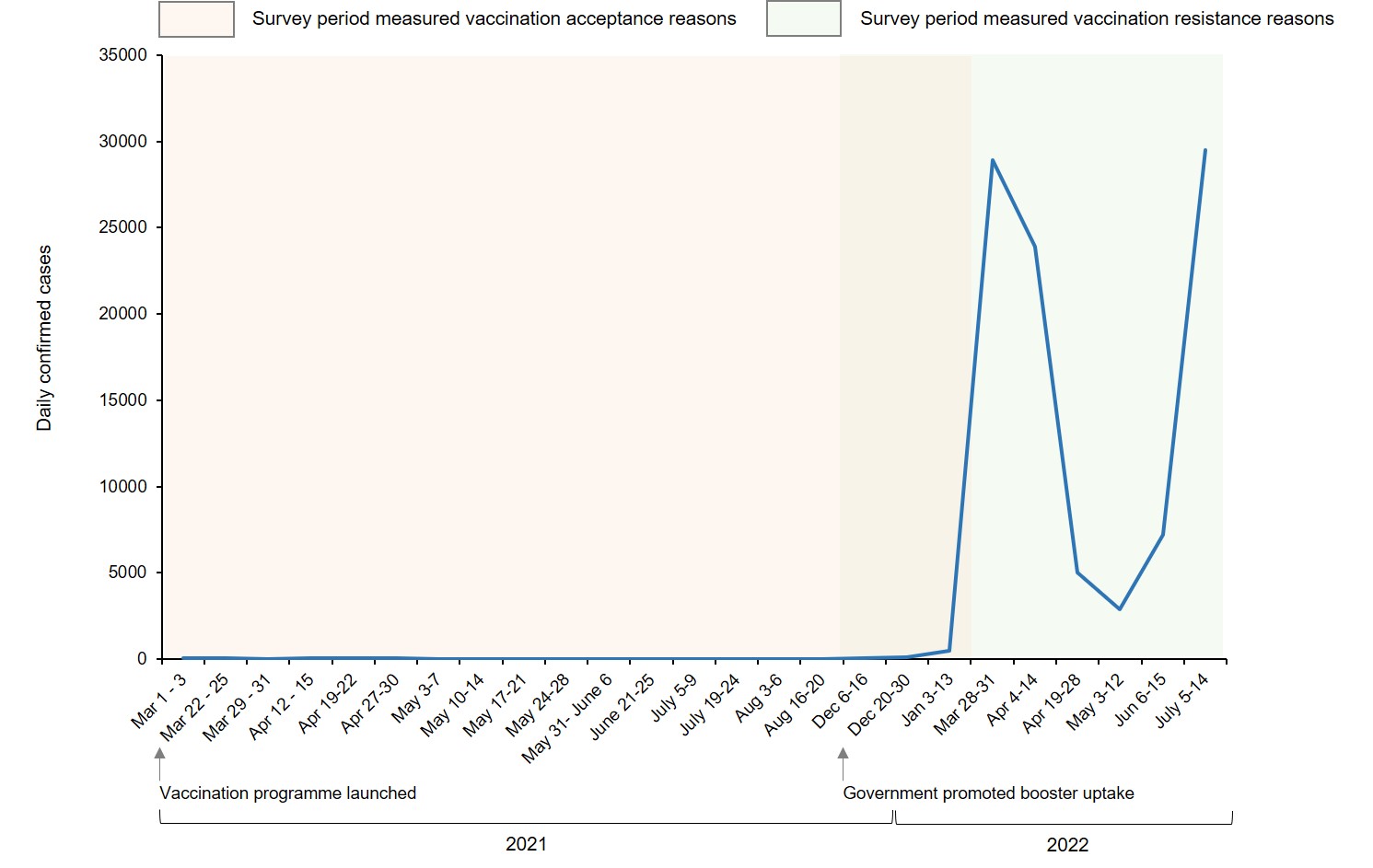

Supplement: Multimedia Appendix 2 [file publichealth_v10i1e50958_app2.docx]
