# Supplementary material for: Motivators and Demotivators for COVID-19 Vaccination Based on Co-Occurrence Networks of Verbal Reasons for Vaccination Acceptance and Resistance: Repetitive Cross-Sectional Surveys and Network Analysis
Source: JMIR Public Health Surveill. 2024 Apr 22;10:e50958. doi: 10.2196/50958 (PMC11074890; doi:10.2196/50958)
Supplement: Multimedia Appendix 3 [file publichealth_v10i1e50958_app3.docx]

Multimedia Appendix 3. Coded categories of reasons for COVID-19 vaccination acceptance and resistance

| Category ^a^ | Examples of verbal reasons |
| --- | --- |
| *For vaccine acceptance* |  |
| Confidence in vaccines | Believe that the vaccine is effective; Always believe that vaccine is the best way to prevent infectious diseases; Believe that the vaccine is safe; Will have milder symptoms even getting infected; Take it earlier the better; Healthy enough to take the vaccine |
| Disease risk | Worry about infecting COVID-19; Worry about infecting mutant strain or other variants of concern; Worry about the outbreaks in neighbouring regions; To stop the spread of virus; Depends on the pandemic situation; For protecting myself; I have chronic diseases/I am old, so taking one is safer |
| Convenience | Have time to take; Because the vaccine is available; I feel convenient to take; Will need to pay for the vaccine later; (if not take the vaccine) I will need to pay for the testing; Worry about no supply of vaccine later; Vaccine is free-of-charge |
| Vaccine mandates | It is compulsory; Being forced by the government; Reduce duration of quarantine; Do not want to do the testing again; Want to visit patients in hospital; Can dine out; Job requirement; Will study abroad; Will need to get vaccinated sooner or later |
| Protecting others | For protecting others; A social responsibility; To achieve herd immunity; For community health; Avoid adding burden to the medial system; A need for everyone to get vaccinated |
| Social norms | To ease family's worries; Family advised me to do so; Doctors' recommendation; Suggested by friends; It is a (behavioural) trend; Other people are taking it; Just follow others |
| Pro-government | To comply with the government; It's time to get vaccinated; Have already taken 2 jabs long time ago, it is the time to take a third dose as the government asked for; To support the government; Responding to the government’s call; Encouraged by the government/trust in government; Believe in the nation |
| Trust in experts | Trust in expert/scientists |
| Back to normal life | To end the pandemic; To see friends; Life can get back to normal as soon as possible; To ease quarantine measures as soon as possible; To ease travel restrictions as soon as possible; To resume economic activity in Hong Kong as soon as possible; To make life more convenient |
| Incentives | Lucky draw; Get vaccination leaves at work; Get free masks |
| *For vaccine resistance* |  |
| Poor health status | Poor physical health; I am too old; I have chronic diseases |
| Lack of vaccine confidence | Concerns about the rapid vaccine development; Insufficient test; The vaccine is too new; Vaccine is useless/ ineffective/ will be infected even after vaccination; Worry about the vaccine quality; Afraid of vaccination/ afraid of pain; Wait for a better vaccine; Wait and see the effectiveness of vaccine first; Do not believe in vaccines |
| Inconvenience | No time to take; Need to queue up, which will put myself at risk; Difficult to travel to the vaccination site |
| Lack of social support | Do not really understand the effect of the COVID-19 virus; Don't know how many doses to take; Do not know how many more vaccines to take; Waiting for others’ advice; No clear information about the vaccine and its side effects; No one to accompany me to take the vaccine |
| Distrust in government | Do not like the government’s approach in pushing vaccination rate; Do not agree with the government’s approach |
| Social norms | Friends and relatives have not been vaccinated/ advised me not to get vaccinated; No one is taking the third jab; Others' opinions against vaccination |
| Vaccine mandates | Not compulsory now; Only will do it if it's compulsory; Do not like being forced to take a vaccine; Not required by the workplace or job yet; No vaccine requirement in other countries |
| Medical preference | Prefer Chinese medicine; Dislike taking vaccination; Should not take vaccine all the time/no need to take every year; Do not want to get vaccine anymore |
| No incentives | Cannot travel; No lucky draw; Depends on the border restriction, Unless the border restriction will be lifted |
| Complacency | Perceived that it is not the good timing to take vaccination; No urgency; Can still wait and see; Observing now; Low chance of infection; This is not a serious outbreak; The pandemic is under control; Depends on the pandemic situation; Depends on the number of cases; Other people get vaccinated will do, I don't need to get it myself; No need to take because already get infected; No need to take because already finished two jabs; No health issues so no need to take; Not sure if it’s needed |

^a^ We excluded 52 responses for the reasons of vaccination acceptance and 23 responses for the reasons of vaccination resistance due to their ambiguous meaning.
