# Supplementary material for: Motivators and Demotivators for COVID-19 Vaccination Based on Co-Occurrence Networks of Verbal Reasons for Vaccination Acceptance and Resistance: Repetitive Cross-Sectional Surveys and Network Analysis
Source: JMIR Public Health Surveill. 2024 Apr 22;10:e50958. doi: 10.2196/50958 (PMC11074890; doi:10.2196/50958)
Supplement: Multimedia Appendix 4 [file publichealth_v10i1e50958_app4.docx]

Multimedia Appendix 4. Mean eigencentrality values of vaccination acceptance and resistance reasons by age groups

| Reasons | 18-24 | 25-34 | 35-44 | 45-54 | 55-64 | 65+ | Overall |
| --- | --- | --- | --- | --- | --- | --- | --- |
| Eigencentrality values of vaccination acceptance reasons | | | | | | | |
| Confidence in vaccines | 0.08 | 0.09 | 0.06 | 0.07 | 0.08 | 0.09 | 0.08 |
| Disease risk | 0.87 | 0.82 | 0.79 | 0.78 | 0.79 | 0.81 | 0.80 |
| Convenience | 0.01 | 0.01 | 0.01 | 0.00 | 0.01 | 0.00 | 0.00 |
| Vaccine mandates | 0.26 | 0.19 | 0.05 | 0.06 | 0.03 | 0.01 | 0.04 |
| Protecting others | 0.36 | 0.51 | 0.59 | 0.61 | 0.60 | 0.57 | 0.58 |
| Social norms | 0.03 | 0.01 | 0.00 | 0.00 | 0.00 | 0.01 | 0.01 |
| Pro-government | 0.03 | 0.01 | 0.01 | 0.01 | 0.01 | 0.02 | 0.01 |
| Trust in experts | 0.00 | 0.00 | 0.00 | 0.00 | 0.00 | 0.00 | 0.00 |
| Back to normal life | 0.20 | 0.16 | 0.13 | 0.12 | 0.12 | 0.09 | 0.12 |
| Incentives | 0.00 | 0.01 | 0.01 | 0.00 | 0.00 | 0.00 | 0.00 |
| Eigencentrality values of vaccination resistance reasons | | | | | | | |
| Poor health status | 0.01 | 0.03 | 0.03 | 0.10 | 0.08 | 0.42 | 0.09 |
| Lack of vaccine confidence | 0.63 | 0.89 | 0.89 | 0.97 | 0.91 | 0.90 | 0.89 |
| Inconvenience | 0.02 | 0.00 | 0.00 | 0.00 | 0.00 | 0.00 | 0.00 |
| Lack of social support | 0.01 | 0.00 | 0.00 | 0.00 | 0.01 | 0.03 | 0.00 |
| Distrust in government | 0.03 | 0.01 | 0.01 | 0.00 | 0.12 | 0.01 | 0.03 |
| Social norms | 0.01 | 0.00 | 0.00 | 0.00 | 0.00 | 0.00 | 0.00 |
| Vaccine mandates | 0.04 | 0.02 | 0.02 | 0.00 | 0.01 | 0.02 | 0.02 |
| Medical preference | 0.00 | 0.00 | 0.00 | 0.00 | 0.02 | 0.00 | 0.00 |
| No incentives | 0.00 | 0.00 | 0.00 | 0.02 | 0.00 | 0.00 | 0.00 |
| Complacency | 0.77 | 0.45 | 0.45 | 0.23 | 0.38 | 0.15 | 0.45 |
